# Differences in biomarkers of cartilage matrix turnover and their changes over 2 years in adolescent and adult volleyball athletes

**DOI:** 10.1186/s40634-017-0081-9

**Published:** 2017-02-23

**Authors:** Heide Boeth, Aoife MacMahon, A. Robin Poole, Frank Buttgereit, Patrik Önnerfjord, Pilar Lorenzo, Cecilia Klint, Anna Pramhed, Georg N. Duda

**Affiliations:** Julius Wolff Institute, Charité - Universitätsmedizin Berlin, Berlin, Germany

**Keywords:** Cartilage, Biomarkers, Volleyball, Athletes, Patient-rated outcomes, Osteoarthritis

## Abstract

**Background:**

This study aimed the feasibility to assess longitudinal changes in biomarkers of cartilage turnover and to determine their relationship with patient-rated outcomes over 2 years in volleyball athletes.

**Methods:**

Thirty-seven athletes were studied: 18 adolescents (age 15.9 ± 0.64 years) in a 2-year intensive volleyball training program and 19 adult recreational volleyball players (age 46.5 ± 4.9 years). Blood and serum samples were taken at baseline (BL) and 2-year follow-up (FU). Subjects completed the International Knee Documentation Committee (IKDC) Subjective Knee Form and the Short-Form 36 (SF-36) at BL.

**Results:**

Thirteen adolescents (72%) had open growth plates at BL (BL open adolescents), the rest had closed growth plates at BL (BL closed adolescents), and all but one adolescent had closed growth plates at FU as assessed by MRI. BL open and closed adolescents had greater levels of the cartilage degradation-based biomarkers 45 mer collagenase peptide of type II collagen (C2C-HUSA) and C-telopeptide of type II collagen (CTX-II) than adults. BL open adolescents showed decreases in C2CHUSA, collagen synthesis marker C-propeptide of type II procollagen (CPII), and CTXII, and adults showed increases in cartilage intermediate layer protein 2 (CILP-2) and C2C-HUSA. In adolescents, IKDC scores were correlated with CPII changes. In adults, SF-36 Physical Component Scores were correlated with cartilage oligomeric matrix protein (COMP) changes.

**Conclusion:**

Significant differences in biomarker levels over time show the feasibility to assess their changes. Greater levels of C2C-HUSA and CTX-II in adolescents than in adults may reflect increased cartilage turnover in response to higher joint loading. CPII and COMP may be more reflective of subjective patient outcomes. These biomarkers may thus be useful in assessing mechanical loading-induced cartilage changes, their associated symptoms, and Osteoarthritis risk in athletes.

## Background

Osteoarthritis (OA) is the most common type of arthritis and causes more functional disability than any other disease (Felson 2014). Primary knee OA is frequently associated with mechanical overloading of joints (Kujala et al. 1994) and is characterized by degradation of the whole joint including cartilage, subchondral bone and surrounding soft tissue (Felson et al. 2000). Previous findings have suggested that high-impact physical activity (Kujala et al. 1994, Kujala et al. 1995, Buckwalter and Lane 1997, Amoako and Pujalte 2014) and/or prior knee injury (Muthuri et al. 2011, Richmond et al. 2013) increase the risk of developing primary OA. However, it is unclear how frequency and intensity of exercise affect human articular cartilage degradation (Hallett and Andrish 1994, Saxon et al. 1999).

Biomarkers of cartilage turnover may reflect structural cartilage changes and thus may identify patients at risk of early onset of OA (Rousseau and Delmas 2007). Previous studies have found that biomarkers of cartilage turnover are altered in athletes after a session of running (Kersting et al. 2005, Niehoff et al. 2011), as well as in varsity collegiate soccer, crew, and swimming athletes after a sports season (O’Kane et al. 2006, Hoch et al. 2012, Mateer et al. 2015). Although past studies have investigated longitudinal changes in biomarkers of cartilage turnover in athletes of a high-impact sport, e.g. soccer, over a sports season (O’Kane et al. 2006, Hoch et al. 2012, Mateer et al. 2015), more long-term changes in such biomarkers in an athletic population remain unclear. Some of these biomarkers may also remain elevated from 1 to 7 years post-injury in athletes who have suffered a knee injury (Ahlen et al. 2015, Kumahashi et al. 2015, Struglics et al. 2015). Additionally, it is important to understand how subjective clinical outcomes are related to changes in biomarkers of cartilage turnover in athletes, which may help determine which biomarkers are most reflective of such patients’ self-reported status.

Long-term changes in biomarkers of cartilage turnover in athletes of a high-impact sport are currently unknown. Moreover, the effects of exercise on cartilage wear in adolescent and adult athletes may be different due to the different developmental stages of these populations. It was previously found that femorotibial cartilage thickness increases in adolescent volleyball players and decreases in adult volleyball players over 2 years (Eckstein et al. 2014). Another study found that 64% of adolescent soccer players display knee abnormalities by Magnetic Resonance Imaging (MRI), particularly bone marrow edema (Soder et al. 2011). Thus, investigating differences between biomarkers of cartilage turnover in adolescent and adult athletes could provide insights into how these biomarkers reflect different cartilage changes and adaptations to exercise in these two populations.

The primary goals of this study were to compare values of and changes in biomarkers of cartilage turnover over 2 years between adolescent and adult volleyball players in order to investigate the roles of growth plate development and volleyball activity on biomarkers of cartilage turnover. The secondary goals were to assess the correlations between baseline (BL) patient-rated outcome measures (PROMs) and longitudinal changes in these biomarkers. Through these goals, we aimed to investigate the roles of volleyball activity and growth plate development on biomarkers of cartilage turnover, which could reflect both exercise-induced and pre-osteoarthritic knee degradation, and also to assess which biomarkers might be clinically relevant. Our hypotheses were that 1) adult volleyball athletes would have higher levels and increases in biomarkers of cartilage degradation than adolescent volleyball athletes, due to the former’s more advanced age and greater lifetime cumulative joint loading; 2) adolescent volleyball athletes would have higher levels and increases in biomarkers of cartilage synthesis due to their continuing growth; and 3) biomarkers of cartilage degradation would be associated with PROMs.

## Methods

### Subjects

This was a prospective, longitudinal cohort study (Level III) which received approval from an Institutional Review Board. A convenience sample of 40 volleyball athletes (20 adolescents and 20 adults) was initially recruited, and all provided written informed consent for participation. For adolescents, inclusion criteria were age less than or equal to 17 years, current participation in the 2-year volleyball training program at Olympiastützpunkt Berlin, and prior volleyball participation in a club for at least 3 years. For adults, inclusion criteria were age greater than 40 years (in order to have an adult cohort that was significantly older than the adolescent cohort and thus would have likely experienced greater joint degradation over time), past participation in the volleyball training program at Olympiastützpunkt Berlin, but with ongoing exclusive and continued volleyball participation for at least 2 h a day, twice a week. Exclusion criteria for both groups were prior knee injury requiring surgery less than 1 year prior to the BL evaluation. The volleyball training program at Olympiastützpunkt Berlin consisted of 2-h training sessions twice daily, 6 days a week, for 2 years.

Two adolescent males with Osgood-Schlatter disease were excluded due to its confounding effects on biomarkers of cartilage turnover and skeletal metabolism (Frantz et al. 2010). One adult male was excluded due to insufficient serum and urine sample volumes. Thus, the final cohort consisted of 37 volleyball athletes, with 18 adolescents and 19 adults (Table 1). Biomarkers of cartilage turnover were evaluated in all subjects at BL and at 2-year follow-up (FU).

**Table 1:** Subject Demographics

	Adolescents	Adults	*P*-value
Age [years] ^a^	15.9 ± 0.64	46.5 ± 4.9	–
Weight [kg] ^a^	76 ± 10.3	82.3 ± 16.2	0.047*
Height [cm] ^a^	188.3 ± 7.8	183.2 ± 8.9	0.081
BMI [kg/m^2^] ^a^	21.4 ± 1.8	24.3 ± 2.9	0.001*
Females ^b^	10 (56)	10 (52)	>0.999
Prior knee injury ^b^	1 (5)	8 (42)	0.0188*

* Statistically significant difference (*P* < 0.05)

^a^ Values expressed as mean ± standard deviation

^b^ Values expressed as *N* (% of age group)

At BL, all subjects were evaluated by a self-reported questionnaire for knee pathology and prior knee injuries that required surgical intervention. One female adolescent had an ACL tear that had been surgically repaired. Two adolescent males and two adolescent females had patellar pain syndrome. Eight adults had prior knee surgery. Among the adult males, one had a lateral meniscectomy, one had lateral and medial meniscectomies and articular cartilage debridement, one had other knee surgery, and one had cartilage debridement and a meniscus surgery. Among the adult females, one had a nerve transection and knee cartilage debridement, one had knee arthroscopy, one had meniscus surgery, and one had a medial meniscectomy. One male adult reported pain below the patella, and two adult females had knee pain. No subjects experienced injuries between BL and 2-year FU. Biomarker levels and patient-rated outcomes were compared between adults with and without prior knee injury to determine how this variable influenced results.

### Sample collection

All blood samples were obtained following an overnight fast and urine samples were collected on the second morning void. Serum and urine samples were collected at both BL and at FU after subjects had received MRI scans and thus had been inactive and lying down for 45 min before being seated for approximately 10 min. Serum samples were obtained from 29 subjects at BL (11 adolescents and 18 adults) and 36 subjects at FU (17 adolescents and 19 adults). Serum samples were insufficient due to a limited amount of blood that had been taken in seven adolescent females and one adult female at BL and in one adolescent male at FU. One adolescent male was missing a urine sample at FU. However, these subjects were included in the analysis as they had adequate samples for at least one time point.

### Patient-rated outcomes

To assess subjective clinical status, all subjects completed the International Knee Documentation Committee (IKDC) Subjective Knee Form and the Short-Form 36 (SF-36) at BL. The IKDC is a validated patient-rated outcome measure that evaluates symptoms, function, and sports activity in patients with various knee problems (Irrgang et al. 2001). The SF-36 is a validated metric for general health outcomes (Patel et al. 2007), and both the Physical Component Score (PCS) and Mental Component Score (MCS) were assessed. For each outcomes measure, scores range from 0 to 100, with a lower score indicating greater disability.

### Magnetic resonance imaging (MRI)

Since the adolescents’ growth status likely affected the levels of biomarkers assessed, the status of their femoral and tibial growth plates was assessed at BL and FU with magnetic resonance imaging (MRI). MRI was performed in a 1.5 T *Avanto* scanner (Siemens Medical Systems, Erlangen, Germany) using a dedicated 8-channel knee coil. The take-off leg was imaged in all participants in supine position with the leg in full extension. A 2D coronal proton-density (PD) weighted turbo spin-echo MR sequence with fat suppression (0.4167×0.4167 mm in-plane resolution, 3 mm slice thickness, 3.6 mm slice spacing, 29 ms echo time, 3520 ms repetition time, 150° flip angle) as well as a 3D axial T2-weighted Multi-Echo Data Image Combination (MEDIC) sequence (0.167×0.167 mm in-plane resolution, 1.2 mm slice thickness, 21 ms echo time, 38 ms repetition time, 8° flip angle) were used for imaging at both BL and FU. All images were assessed on a picture archiving communications system (PACS) by a radiologist with 12 years of experience in musculoskeletal imaging. The radiologist was blinded to the clinical information of each subject and to the time point of the MRI.

### Biomarker analyses

Serum and urine samples were immediately stored at −70 °C until testing. After being frozen on dry ice, they were transported for assay to AnaMar AB (Lund, Sweden) and IBEX Pharmaceuticals (Montreal, Quebec, Canada).

The serum cartilage molecular biomarkers studied were the collagen degradation markers cartilage oligomeric matrix protein (COMP), cartilage intermediate layer protein 2 (CILP-2), and serum type II collagen cleavage neoepitope (sC2C), as well as the collagen synthesis marker C-propeptide of type II procollagen (CPII). COMP is expressed as U/L, and CILP-2, sC2C, and CPII are expressed as ng/mL.

The urine cartilage molecular biomarkers studied were the collagen degradation markers 45-mer collagenase-generated peptide of human type II collagen (C2C-HUSA) and CTX-II, which are expressed as ng/mmol of creatinine. At IBEX, all the samples were tested in duplicate for all assays (C2C, CPII, C2C-HUSA and CTX-II and urine creatinine). At AnaMar AB, all the samples were tested in duplicate for COMP and CILP-2. All study subjects were randomly assigned to the assay plates regardless of which group they belonged to. Both time points for each subject were always assayed on the same plate.

#### Serum collagen degradation biomarkers

COMP was measured using a sandwich enzyme-linked immunosorbent assay (ELISA) (AnaMar AB) and the cartilage collagen degradation biomarker C2C (collagenase generated carboxy-terminal neoepitope of type II collagen) using a competitive inhibition ELISA (IBEX). CILP-2 was assayed using an in-house research competitive ELISA (AnaMar AB).

#### Serum collagen synthesis biomarkers

CPII (type II procollagen carboxy-propeptide) was assayed using a competitive inhibition ELISA (IBEX).

#### Urine type II collagen degradation biomarkers

Two biomarkers of degradation were measured in urine samples: CTX-II (C-telopeptide of type II collagen) using a competitive assay (CartiLaps; IDS) and C2C-HUSA (collagenase-generated peptides of human type II collagen) using a sandwich assay (IBEX) (Cibere et al. 2009). Creatinine was measured at IBEX using an enzymatic colorimetric kit (QuantiChrom™; BioAssay Systems, Hayward, CA, USA).

#### Biomarker assays

The assay details, reproducibility (intra and inter assay variation) and accuracy of all these assays (recovery of added analyte) are described in detail on the websites of the manufacturers (interassay variability: 2–3%, intraassay variability: 2–4%, recovery: 93–116%) with the exception of the competitive CILP-2 assay which is as follows: Microtitre plates (Costar high binding 9018) were coated with a 61 amino acid long synthetic polypeptide from the CILP2 domain 1 (V551-D611 (UniProt Q8IUL8) Schafer-N, Copenhagen, Denmark). Plates were incubated at room temperature overnight. Plates were washed with PBS-Tween (PBS-T, Medicago) four times and incubated with block solution (1% Probumin from Millipore in PBS-T) for 2–3 h. Standard (range 100 ng/ml-0.14 ng/ml) and athlete serum samples were diluted 1:10 in conjugate buffer (Medicago 25-0142+ 0.1 mg/ml goat IgG from Sigma) and pre-incubated with HRP-coupled anti-CILP-2 antibody (polyclonal affinity-purified goat antibody (Capra Science, Sweden) against the coating CILP-2 peptide) on non-binding plates (U96 PP 0.5 ml, Natural, Thermo Scientific) for 1 h. The blocked plates were washed with PBS-T four times and the pre-incubated sample was transferred to the freshly washed plates followed by further incubation at room temperature for 2 h. After washing with PBS-T (6 times) TMB substrate (Medicago) was added and the signal allowed developing for 20 min protected from light. The reaction was stopped by the addition of 0.5 M H2SO4 before reading the plates at 450 nm. Samples were analysed in duplicate (patient samples from BL and FU were run on the same plate). The CILP-2 assay standard, a 16 kDa protein (His6tagged aa C206-P246 merged with V551-D611) was produced in E. coli and IMAC/Ni2+ column purified. Standard curve was produced by spiking standard protein to non-detecting CILP-2 serum. The CILP-2 competitive assay had an intra assay variation of 11.1% and the inter assay variations for high control was 20.7% and for low control 22.2%. All analyses were carried out without any prior knowledge of the individual samples.

### Statistical analysis

Quantitative variables are expressed as means and 95% confidence intervals unless stated otherwise. Data were assessed for normality using the Shapiro-Wilk test. Demographic variables and patient-rated outcome scores were normally distributed, whereas biomarker data were not. Adolescents were stratified by BL growth plate status (open or closed) for biomarker comparisons. Differences in BL and FU biomarker levels between the three groups (BL open adolescents, BL closed adolescents, and adults) were assessed with Kruskal Wallis tests, with pairwise post-hoc analyses when significant adjusted for multiple testing. Differences in biomarker levels between adults with and without prior knee injuries were assessed with Mann Whitney U tests. As there was only one adolescent with prior knee injury, no comparison in biomarkers was made between adolescents with and without prior knee injury. Differences in demographic variables and patient-rated outcomes were assessed with Student’s *t*-tests. Longitudinal changes in biomarkers were assessed with Wilcoxon Signed Rank tests. Correlations between patient-rated outcomes and longitudinal biomarker changes were assessed with Spearman’s Rank Correlations. An *r*-value of 0.1 to 0.39 was considered a weak correlation, an *r*-value of 0.4 to 0.6 was considered a moderate correlation, and an *r*-value of 0.61 to 1.0 was considered a strong correlation. A one-way ANOVA was used to compare IKDC and SF-36 scores between BL closed adolescents, BL open adolescents, and adults. All statistical tests were conducted using SPSS Statistics 18 (IBM SPSS Statistics, USA) with a significance level of 0.05.

## Results

### Patient-rated outcomes

At BL, adults had a mean IKDC score of 94.3 (range, 75.8 to 100.0) and a mean SF-36 PCS of 53.4 (range, 47.5 to 56.9). Adolescents had a mean IKDC score of 93.9 (range, 70.1 to 100.0) and a mean SF-36 PCS of 55.5 (range, 36.6 to 65.9). There were no significant differences found in IKDC score (*p* = 0.885) or SF-36 PCS (*p* = 0.193) between age groups. Adults with prior knee injuries had significantly worse SF-36 PCS than adults without prior knee injuries (52.4 [50.8, 54.1] vs. 54.9 [53.3, 56.4], *p* = 0.033). The comparison of IKDC and SF-36 scores between BL closed adolescents, BL open adolescents, and adults showed no significant differences between the groups (*P* ≥ 0.145).

### Growth plate status

At BL, 13 of the 18 adolescents (72%) had open tibial and femoral growth plates, and were classified as BL open adolescents for all analyses, and two boys and three girls had closed growth plates, and were classified as BL closed adolescents for all analyses. At FU, all adolescents, except for one male, had closed growth plates.

### Biomarkers

**Fig. 1:**
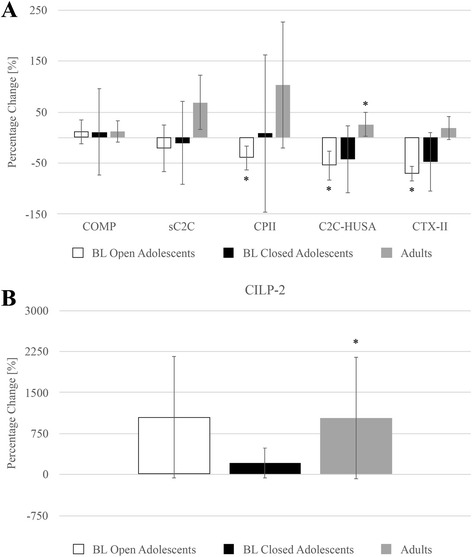
Percentage longitudinal changes in biomarkers in *baseline* open adolescents, *baseline closed adolescents* and adults. *Error bars* represent 95% confidence intervals. *P < 0.05 for change from baseline to follow-up. **a** Percentage longitudinal changes in all biomarkers and ratios assessed, with the exception of *cartilage intermediate layer protein 2 (CILP-2)*. **b** Percentage longitudinal change in *cartilage intermediate layer protein 2 (CILP-2)*

BL and FU biomarker values are shown in Table 2. At both BL and FU, C2C-HUSA was greater in BL open adolescents than in adults (*p* < 0.001). At BL, C2C-HUSA was also greater in BL closed adolescents than in adults (*p* = 0.007) but not at FU (*p* = 0.112). At both BL and FU, CTX-II was greater in BL open adolescents than in adults (*p* < 0.001). CTX-II was also greater in BL closed adolescents than in adults at BL (*p* = 0.030) and FU (*p* = 0.034). Longitudinal changes in biomarkers are shown in Table 3 and Fig. 1. BL open adolescents showed decreases in CPII (*p* = 0.028), C2C-HUSA (*p* = 0.012), and CTX-II (*p* = 0.002). BL closed adolescents showed no changes in biomarker levels from BL to FU (*p* ≥ 0.080). Adults exhibited increases in CILP-2 (*p* = 0.021) and C2C-HUSA (*p* = 0.033) from BL to FU. There were no significant differences found in biomarker levels at BL or FU between adults with and without prior knee injuries (*p* ≤ 0.206).

**Table 2:** Biomarker Values ^a^

		BL Open Adolescents	BL Closed Adolescents	Adults	*P*-value
CILP-2 [ng/ml]	BL	0.56 (−0.059, 1.2)	0.98 (0.60, 1.4)	1.4 (0.90, 2.0)	0.13
	FU	2.2 (1.4, 3.1)	3.0 (1.3, 4.6)	2.6 (2.0, 3.2)	0.775
COMP [U/l]	BL	15.2 (11.8, 18.5)	11.5 (3.7, 19.3)	12.8 (10.8, 14.8)	0.406
	FU	15.0 (13.1, 16.8)	12.1 (6.7, 17.6)	13.4 (11.8, 15.0)	0.537
sC2C [ng/ml]	BL	167.9 (108.6, 227.1)	137.5 (18.6, 256.4)	120.5 (90.3, 150.7)	0.177
	FU	123.3 (105.8, 140.7)	113.4 (70.2, 156.6)	159.6 (120.0, 199.3)	0.241
CPII [ng/ml]	BL	310.4 (212.8, 407.9)	177.2 (−5.1, 359.5)	199.3 (139.7, 258.9)	0.164
	FU	176.9 (139.7, 214.1)	161.8 (42.5, 281.0)	262.9 (144.7, 381.0)	0.862
C2C-HUSA [ng/mmol]	BL	7.4 (2.3, 12.6)	3.6 (0.21, 7.0)	0.43 (0.33, 0.53)	<0.001 ^*^ 0.007 ^**^
	FU	3.2 (1.9, 4.5)	1.3 (0.53, 2.0)	0.54 (0.39, 0.69)	<0.001 ^*^ 0.112 ^**^
CTX-II [ng/mmol]	BL	10,675.6 (6038.9, 15,312.3)	3710.8 (259.7, 7161.9)	255.4 (177.2, 333.7)	<0.001 ^*^ 0.030 ^**^
	FU	3312.2 (2255.6, 4368.9)	1499.8 (1181.6, 1818.1)	281.1 (203.5, 358.7)	<0.001 ^*^ 0.034 ^**^

^*^ Statistically significant difference between BL open adolescents and adults

^**^ Statistically significant difference between BL closed adolescents and adults

^a^ *BL* baseline, *FU* follow-up, *BL Open Adolescents* adolescents with open growth plates at BL, *BL Closed Adolescents* adolescents with closed growth plates at BL. Data provided as mean (95% confidence interval). *P*-values refer to differences between the three groups at BL or FU

**Table 3:** Biomarker Longitudinal Changes ^a^

	BL Open Adolescents	*P*-value	BL Closed Adolescents	*P*-value	Adults	*P*-value
CILP-2 [ng/ml]	0.79 (−0.32, 1.9)	0.116	1.8 (−0.72, 4.4)	0.144	1.2 (0.23, 2.1)	0.021*
COMP [U/l]	1.3 (−1.4, 4.1)	0.917	0.13 (−8.5, 8.7)	>0.999	0.44 (−1.4, 2.3)	0.948
sC2C [ng/ml]	−54.3 (−131.4, 22.7)	0.116	−36.3 (−142.6, 70.1)	0.273	43.1 (−14.4, 100.7)	0.17
CPII [ng/ml]	−128.8 (−231.4, −26.2)	0.028*	−57.9 (−253.1, 137.2)	0.273	73.4 (−75.1, 221.8)	0.586
C2C-HUSA [ng/mmol]	−7.8 (−17.8, 2.0)	0.012*	−2.5 (−6.7, 1.6)	0.080	0.1079 (−0.0061, 0.22)	0.033*
CTX-II [ng/mmol]	−11,051.3 (−18,920.3, −3182.2)	0.002*	−2652.8 (−7578.2, 2272.7)	0.080	25.6 (−18.5, 69.9)	0.26

*Statistically significant difference (*P* < 0.05)

^a^ *BL* baseline, *BL Open Adolescents* adolescents with open growth plates at BL, *BL Closed Adolescents* adolescents with closed growth plates at BL. Data provided as mean (95% confidence interval)

### Correlations between patient-rated outcomes and longitudinal biomarker changes

**Fig. 2:**
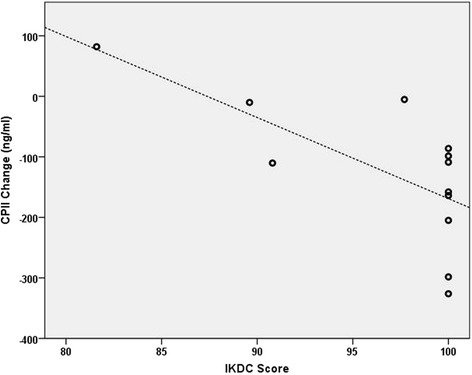
Adolescent *collagen synthesis marker C-propeptide of type II procollagen (CPII)* changes plotted against baseline *International Knee Documentation Committee* scores. *N* = 10 due to seven adolescent females missing baseline serum samples and one adolescent male missing a follow-up serum sample

**Fig. 3:**
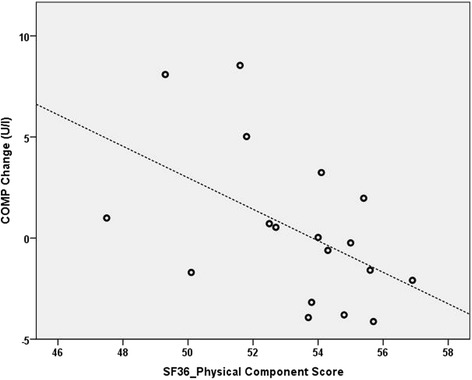
Adult *cartilage oligomeric matrix protein (COMP)* changes plotted against *Short-Form 36 (SF36)* Physical Component Scores. *N* = 18 due to one adult female missing a baseline serum sample

Results of correlation analyses are shown in Tables 4 and 5. In adolescents, BL IKDC scores were found to be significantly and strongly correlated with CPII longitudinal changes (*r* = −0.662, *p* = 0.037; Fig. 2). In adults, BL SF-36 PCS were found to be significantly and moderately correlated with COMP longitudinal changes (*r* = −0.519, *p* = 0.027; Fig. 3).

**Table 4:** Spearman’s Correlations Between Baseline Patient-Rated Outcomes and Longitudinal Biomarker Changes in Adolescents ^a^

		IKDC	SF-36 PCS
CILP	*r*	0.307	−0.212
	*P*-value	0.388	0.556
COMP	*r*	−0.587	0.127
	*P*-value	0.074	0.726
sC2C	*r*	−0.498	−0.236
	*P*-value	0.143	0.511
CPII	*r*	−0.662	−0.248
	*P*-value	0.037*	0.489
C2C-HUSA	*r*	−0.027	0.248
	*P*-value	0.940	0.489
CTX-II	*r*	−0.464	−0.018
	*P*-value	0.176	0.960

*Statistically significant difference (*P* < 0.05)

^a^ *N* = 10 for all correlation analyses due to one adolescent male and five adolescent females missing baseline serum samples, and one adolescent male missing a follow-up serum sample

**Table 5:** Spearman’s Correlations Between Baseline Patient-Rated Outcomes and Longitudinal Biomarker Changes in Adults ^a^

		IKDC	SF-36 PCS	SF-36 MCS
CILP	*r*	−0.099	−0.296	−0.148
	*P*-value	0.695	0.233	0.559
COMP	*r*	0.114	−.519	0.149
	*P*-value	0.653	0.027*	0.556
sC2C	*r*	−0.319	0.406	0.163
	*P*-value	0.197	0.095	0.518
CPII	*r*	−0.293	0.207	0.232
	*P*-value	0.239	0.409	0.354
C2C-HUSA	*r*	0.193	0.130	−0.136
	*P*-value	0.428	0.596	0.578
CTX-II	*r*	−0.195	0.228	−0.395
	*P*-value	0.425	0.348	0.094

^a^ *N* = 18 for all correlation analyses due to one adult female missing a baseline serum sample, except for C2C-HUSA

## Discussion

The primary aims of this study were to compare biomarkers of cartilage turnover in adolescent and adult volleyball athletes over 2 years. Our main findings were that in adolescents, regardless of growth plate status, there were higher levels of C2C-HUSA and CTX-II than in adults. Adolescents with BL open growth plates showed decreases in CPII, C2C-HUSA, and CTX-II, whereas adults showed increases in C2C-HUSA and CILP-2, and adolescents with closed BL growth plates showed no biomarkers changes from BL to FU. IKDC scores were correlated with CPII changes in adolescents, whereas SF-36 scores were correlated with COMP changes in adults.

C2C-HUSA is an assay that recognizes a 45 mer collagenase-generated cartilage derived peptide containing the C2C neoepitope. It has been found to increase with the onset and progression of cartilage degeneration in patients with early articular cartilage degeneration and in those with knee OA (Cibere et al. 2009). A study in knee OA patients found that C2C-HUSA levels were correlated with more severe knee cartilage lesions, lower Knee Injury and Osteoarthritis Outcome Scores (KOOS), and worse functional abilities of the lower limb (Tamm et al. 2014). The increase of C2C-HUSA we found in adults is likely reflective of cartilage breakdown in that cohort over 2 years, corroborating a previous study in this cohort which found a decrease in their knee cartilage thickness and subchondral bone plate area during this time period (Eckstein et al. 2014). In contrast, C2C-HUSA decreased in BL open adolescents likely due to their ongoing growth, in which type II collagen cleavage by collagenases is very pronounced in the hypertrophic zone (Bernardo et al. 2011). Interestingly, both adolescents with and without open growth plates at BL had significantly higher levels of C2C-HUSA at BL than adults, although this did not reach statistical significance at FU for BL closed adolescents. This difference between the age groups is possibly due to the higher volleyball training intensity in the adolescents in comparison to the adults, which may have led to a higher rate of immediate cartilage degradation in the adolescents, and suggests that C2C-HUSA may reflect more short-term, immediate cartilage turnover stimulated by high joint loading. This encourages further studies of C2C-HUSA to determine whether it can predict risk of knee OA in this high-risk population.

High levels of the articular cartilage degradative marker CTX-II, the C-telopeptide of type II collagen, have also previously been associated with greater risk (Cibere et al. 2009) and severity (Fang et al. 2001, Jiao et al. 2016) of radiographic OA, as well as with MRI-detected knee cartilage defects in subjects with and without radiographic OA (Ding et al. 2005). Elevated CTX-II has also been found in the urine of growing children, attributed to growth plate activity, and was found to decrease with age in a study of healthy adults between the ages of 20 and 55 years (Mouritzen et al. 2003). These findings could explain the significantly higher levels of CTX-II in both groups of adolescents than in adult volleyball players in our study at both BL and FU and the decrease in CTX-II from BL to FU in the BL open adolescents, reflecting the transition from open to closed growth plates. However, our finding that levels of CTX-II at FU were greater in both groups of adolescents than in adults, when all adolescents but one had closed growth plates, suggests that CTX-II also reflects elevated cartilage turnover due to the adolescents’ higher volleyball activity levels in addition to growth. Similarly, a study by O’Kane at al. in varsity college athletes found that CTX-II was significantly higher in runners than in crew, swimmers, or controls (O’Kane et al. 2006). They concluded that the high levels of CTX-II in runners were caused by the increased amount of cartilage remodeling or degradation in that group due to joints stressed by high amplitude loading of the lower skeleton (O’Kane et al. 2006). Volleyball players likely experience similar amounts of mechanical loading as runners, as both are high-impact sports with substantial loading of the lower limbs. Thus, this study adds to previous findings suggesting that CTX-II reflects mechanical loading-induced cartilage turnover, making it a promising biomarker to assess pre-OA cartilage changes in an athletic, high-risk population.

CILP-2 is homologous to CILP-1, cartilage intermediate layer protein-1, which is expressed in the intermediate zone of articular cartilage and has been associated with cartilage degenerative diseases (Lorenzo et al. 1998). CILP-2 is more localized to the deeper intermediate zone of articular cartilage at maturity, and its expression has been found to be downregulated in mice with experimentally-induced degenerative OA (Bernardo et al. 2011). One study also found CILP-2 to be decreased in adults with radiographic knee OA compared to healthy controls. These findings suggest that CILP-2 is inversely associated with cartilage degeneration. Moreover, CILP-2 was found to be restricted to articular cartilage and the meniscus and absent in growth plate cartilage, suggesting that it is characteristic of the specialized composition of the extracellular matrix synthesized by articular chondrocytes (Bernardo et al. 2011). Thus, the longitudinal increase in CILP-2 seen in adults may reflect long-term articular cartilage remodeling from biomechanical stresses augmented by volleyball participation. The lack of longitudinal differences in CILP-2 in the adolescents suggests that there are other age-related factors that contribute to its changes over time. It is also interesting that there were no significant differences in BL or FU levels of CILP-2 found between adolescents, either combined or stratified by BL growth plate status, and adults, suggesting that levels of this biomarker may fluctuate over time in response to remodeling of articular cartilage. Since CILP-2 has scarcely been investigated in the literature, further studies are required to assess how it reflects cartilage changes in athletic as well as osteoarthritic populations.

COMP, cartilage oligomeric matrix protein, is a non-collagenous protein which binds and stabilizes type II collagen fibers and is found in synovium, ligamentous tissue, tendon, meniscus, and particularly articular cartilage (Hoch et al. 2012). In adults, SF-36 PCS scores were moderately correlated with COMP changes. We found that adults with prior knee injuries had significantly worse SF-36 PCS than those without prior knee injuries, although there was no difference in COMP levels found between these two groups. Previous studies have found serum COMP to increase in runners after a marathon (Neidhart et al. 2000, Kim et al. 2009), but another found that increased COMP in college soccer players over a season did not reach the calculated minimum detectable change (Hoch et al. 2012). Kersting et al. found that COMP levels did not significantly change after a 1-h training session, but noted that the standard deviation and range of COMP levels were notably increased post-exercise, indicating that changes may have occurred, albeit in different directions (Kersting et al. 2005). The COMP levels in our adult volleyball players also ranged widely at each point, with ranges of 14.1 U/I and 12.4 U/I at BL and FU, respectively. This may be why we did not find the COMP level to statistically significantly change over time in the adults, although there were enough changes to correlate with SF-36 PCS scores. This suggests that adult volleyball athletes with worse overall physical status, regardless of prior injury status, tended to have increases in COMP over 2 years. Although the causes for this are unclear, larger studies with longer follow-up times are required to assess how COMP reflects patient-rated outcomes and cartilage changes in high-risk populations.

This study had several limitations. A number of subjects were missing serum samples at either BL or FU, which decreased our sample size. Many subjects also had prior knee injuries; however, we aimed to assess how biomarkers of cartilage turnover were affected over time in active volleyball athletes, including those who had recovered from prior knee injuries. We also compared biomarker values and patient-rated outcomes between these subgroups in the adults. Patient-reported outcomes were not collected at FU, so longitudinal changes in this measure could not be assessed.

There were also strengths to this study. Our cohort consisted of two highly uniform age groups with different levels of volleyball participation for comparison. Biomarkers were assessed from both serum and urine samples, which provided a comprehensive assessment of metabolic and pathophysiological cartilage changes. Patient-rated outcomes in both a knee-specific and general health score were used to assess subjective clinical outcomes at BL, which provided a more holistic picture of subjective clinical status. This is also the first study to assess long-term changes in biomarkers of cartilage turnover in adolescent and adult volleyball athletes.

### Conclusions

In conclusion, we found that the assessment of different biomarker levels and their changes over time is feasible in adolescent volleyball athletes with both open and closed growth plates, even though they still grow, and adult volleyball athletes. Specifically, adolescent volleyball athletes with both open and closed growth plates had higher levels of the type II collagen degradative biomarkers C2C-HUSA and CTX-II at BL than adult volleyball athletes. Further, we found that CPII, C2C-HUSA, and CTX-II decreased in BL open adolescents, and that C2C-HUSA and CILP-2 increased in adult volleyball athletes over 2 years. These findings suggest that these biomarkers reflect differences in cartilage responses to high-impact joint loading in athletes of different ages and stages of development. The associations between patient-rated outcomes with CPII and COMP changes suggest that these biomarkers may reflect patients’ subjective clinical status. Future studies are required to assess how these biomarkers relate to the risk of future development of OA in athletes.

## Abbreviations

ACL: Anterior Cruciate Ligament
BL: Baseline
C2C-HUSA: Cartilage degradation-based biomarkers 45 mer collagenase peptide of type II collagen
CILP-2: Cartilage intermediate layer protein 2
COMP: Cartilage oligomeric matrix protein
CPII: Collagen synthesis marker C-propeptide of type II procollagen
CTX-II: C-telopeptide of type II collagen
FU: 2-year follow-up
IKDC: International Knee Documentation Committee
MCS: Mental Component Score
MEDIC: Multi-Echo Data Image Combination
MRI: Magnetic Resonance Imaging
OA: Osteoarthritis
PACS: Picture archiving communications system
PCS: Physical Component Score
PD: Proton-density
sC2C: Serum type II collagen cleavage neoepitope
SF-36: Short-Form 36
